# Malignant Epiretinal Membrane After Proton Beam Radiation of Ciliary Body Melanoma

**DOI:** 10.18502/jovr.v18i4.14558

**Published:** 2023-11-30

**Authors:** Arman Mashayekhi, Mohammad Yousaf, Syril Dorairaj, Kevin Wu, Isabella Wagner, James Bolling

**Affiliations:** ^1^Mayo Clinic Florida, Ophthalmology Department, FL, USA; ^2^Mayo Clinic Florida, Pathology Department, FL, USA

**Keywords:** Uveal Melanoma; Proton Beam Radiation, Uveal Melanoma Seeding; Vitreous Seeding, Epiretinal Seeding; Malignant Epiretinal Membrane

## Abstract

**Purpose:**

To report the development of malignant epiretinal membrane after radiation of ciliary body melanoma.

**Case Report:**

A 65-year-old woman was referred for evaluation of a ciliary body tumor in her right eye. On examination, a pigmented ciliary body tumor, displacing the iris anteriorly, was visible superotemporally and ultrasound biomicroscopy revealed a large solid ciliary body tumor. She was diagnosed with ciliary body melanoma and treated with proton beam radiation. Over the following 29 months, the treated tumor regressed but optical coherence tomography (OCT) showed the development of a dense epiretinal membrane. Enucleation was performed and histopathological examination showed viable melanoma cells in the vitreous cavity with sheet-like growth of viable spindle melanoma cells on the epiretinal surface.

**Conclusion:**

The development of a pigmented epiretinal membrane in eyes with uveal melanoma should raise the possibility of a malignant epiretinal membrane.

##  INTRODUCTION 

Diagnosis of uveal melanoma is usually based on the presence of typical clinical findings, and the most common treatment modality for small and medium-sized tumors is radiation. The presence of ophthalmoscopically-visible pigment in the vitreous cavity of eyes harboring UM has been reported and is believed to represent benign pigment-laden macrophages in most cases.^[1]^

In some cases, however, the vitreous pigment has been shown to represent seeds of viable melanoma cells.^[1,2,3]^ Such vitreous seeding is thought to develop after the tumor causes a rupture in the overlying Bruch membrane and the malignant melanoma cells gain access to the vitreous cavity after invading the neurosensory retina. Vitreous seeding by UM has been reported in treatment-naive eyes or after interventions such as needle biopsy, radiation, or endoresection.^[2,3]^

We report a case of vitreous and epiretinal seeding after proton beam radiation of ciliary body melanoma. Histopathological evaluation after enucleation confirmed the presence of malignant melanoma cells in the vitreous cavity and on the surface of the retina, forming a malignant epiretinal membrane.

**Figure 1:**
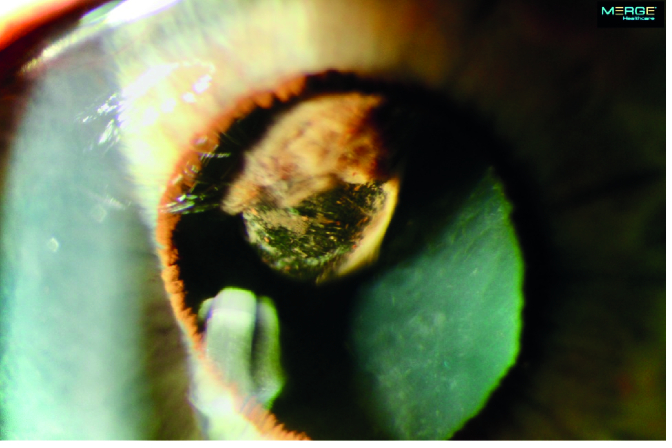
At presentation, slit lamp photograph of the right eye shows a pigmented ciliary body tumor superotemporally with a cavitary space over its apical portion.

**Figure 2:**
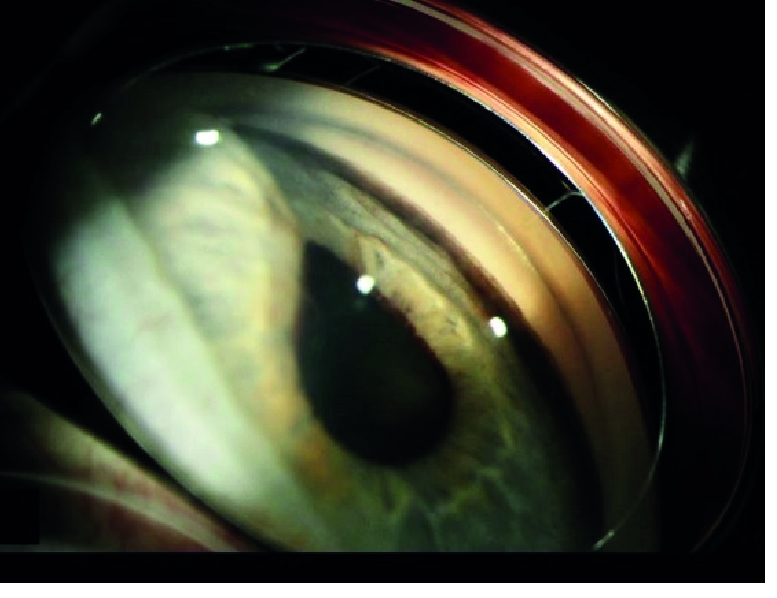
Gonio photograph of the right eye at presentation shows local bulging forward of the iris over the ciliary body tumor. Note the flat heavy pigmentation of the trabecular meshwork.

**Figure 3:**
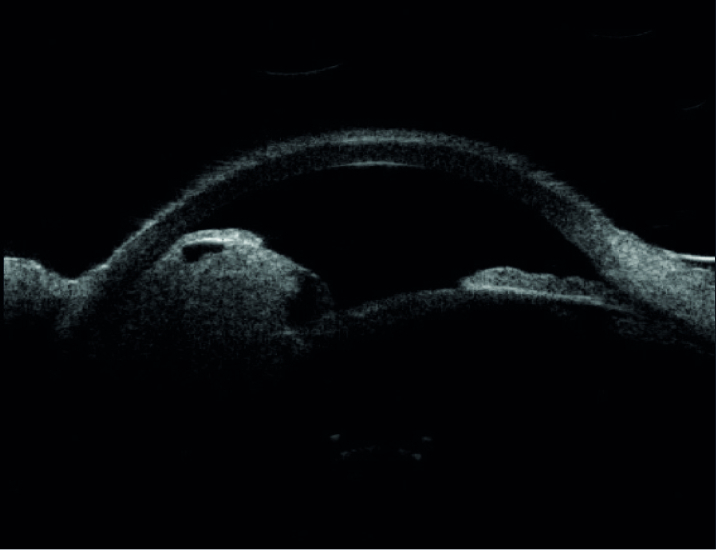
Ultrasound biomicroscopy of the right eye at presentation reveals the presence of a solid ciliary body tumor causing anterior displacement of the overlying iris. Note the presence of a cavitary space over the apex of the tumor.

**Figure 4:**
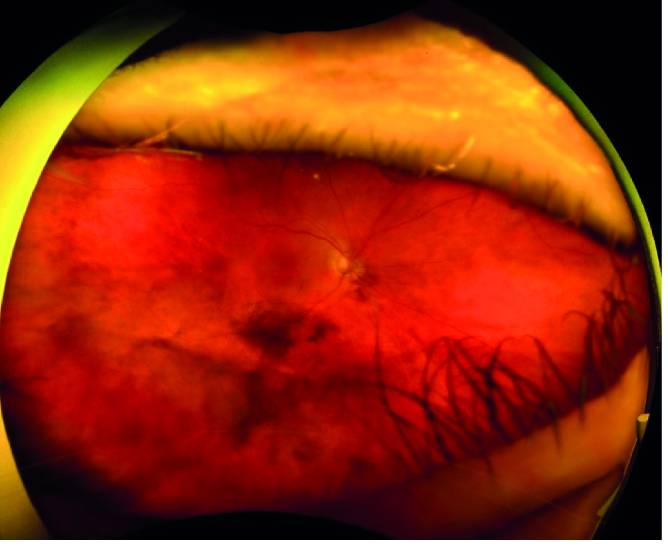
Ultrawide angle image obtained 16 months after proton beam radiation, shows vitreous pigmentation inferiorly. There is also an area of flat pigmentation over the retinal surface along the inferior vascular arcade.

**Figure 5:**
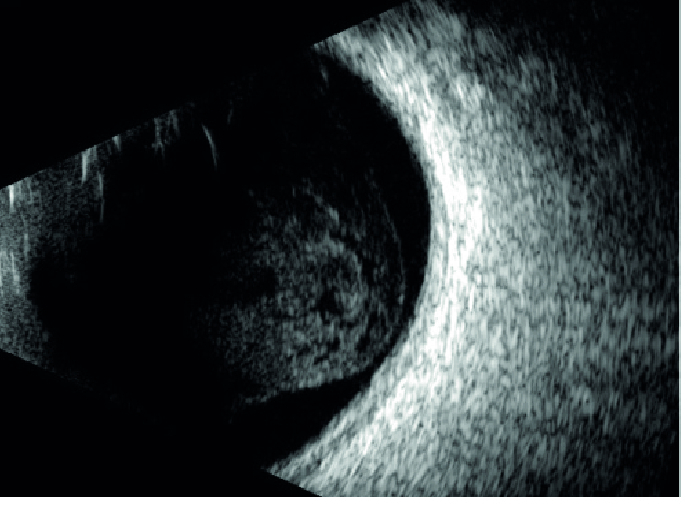
B-scan ultrasound 16 months after proton beam radiation shows complete posterior vitreous detachment and moderate point-like echodensities in the posterior vitreous cavity.

**Figure 6:**
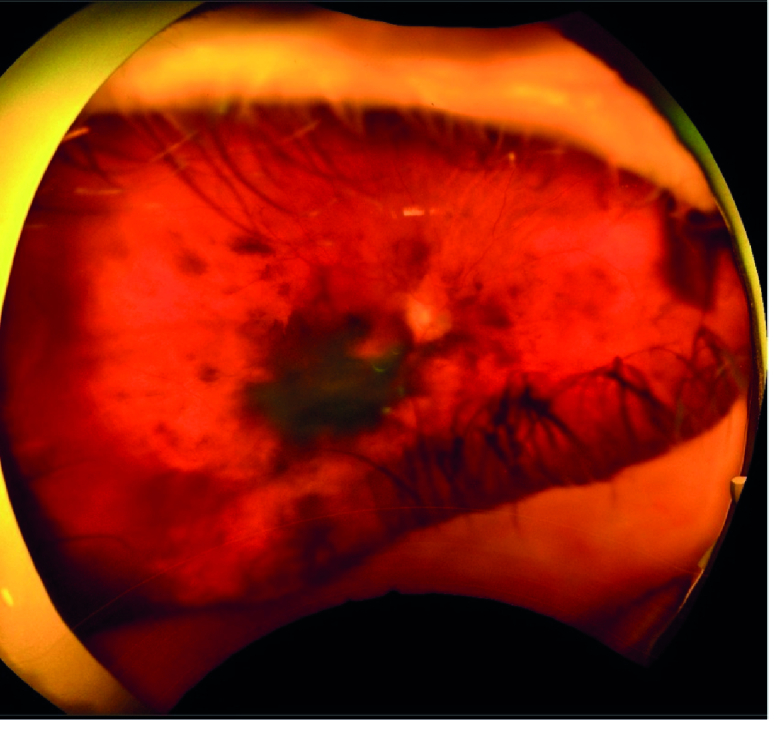
Optos image obtained 26 months after proton beam radiation shows increased vitreous pigmentation and an area of heavy epiretinal pigmentation over the central and inferior macular area.

**Figure 7:**
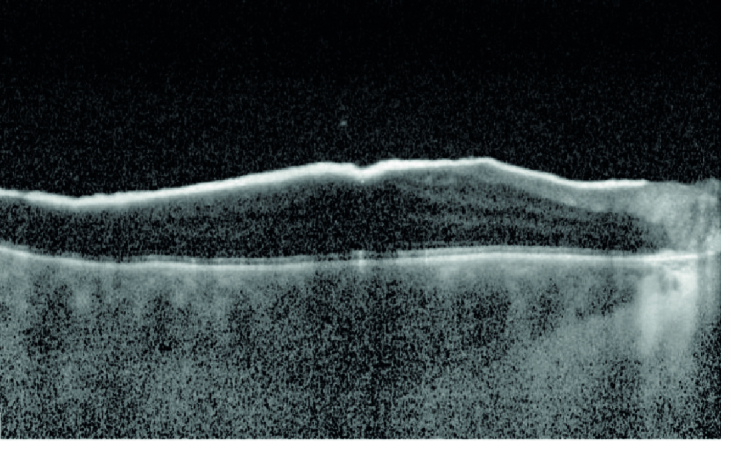
Optical coherence tomography (OCT) image of the right eye 26 months after proton beam radiation shows a relatively thick optically dense epiretinal membrane in the macular area.

**Figure 8:**
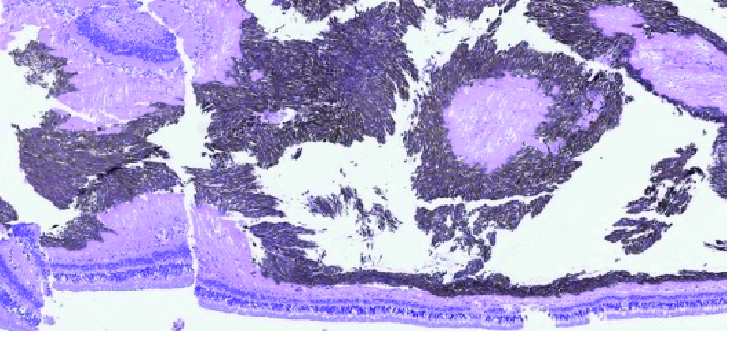
Histopathological image of the right eye after enucleation shows clusters of pigmented melanoma cells in the vitreous cavity. Note the membrane-like growth of pigmented spindle melanoma cells on the surface of the neurosensory retina (Hematoxylin-Eosin stain).

##  CASE REPORT 

A 65-year-old woman experienced a sudden onset of severe pain in her right eye. An examination by her local ophthalmologist revealed high intraocular pressure (IOP) and a pigmented mass in her right eye. She was started on glaucoma eye drops and was referred to the ophthalmology department at Mayo Clinic for further management. On presentation, the best-corrected visual acuity was 20/80 in the right eye and 20/25 in the left eye. The IOPs were 62 mm Hg and 20 mm Hg in the right and left eyes, respectively. Slit lamp examination showed a large pigmented ciliary body tumor superotemporally in the right eye that had caused local anterior displacement of the iris without any visible anterior chamber invasion [Figure 1]. On gonioscopy, diffuse flat pigmentation of the trabecular meshwork was visible for 12 hr; moreover, no anterior chamber or angle invasion by the tumor was observed [Figure 2]. Ultrasound biomicroscopy revealed a solid ciliary body tumor with anterior displacement of the iris [Figure 3].

A diagnosis of ciliary body melanoma was rendered, and the tumor was treated with 60 Gy of proton radiation in 4 fractions. During the placement of tantalum fiducial markers, cyclocryotherapy was also performed to manage the uncontrolled high IOP. As per the patient's request, a needle biopsy of the tumor for prognostic genetic testing was not performed. The patient developed rapid progression of cataract soon after completion of proton beam radiation and underwent cataract surgery one month later. During the surgery, a portion of the anterior lens capsule was excised and submitted for histopathological evaluation that revealed no malignant melanoma cells.

Sixteen months after proton radiation the treated ciliary body melanoma showed signs of regression, but mild degrees of pigment dusting were visible in the inferior vitreous cavity on ophthalmoscopy. In addition, flat deposits of pigment were noticeable on the surface of the retina in the macular area [Figure 4]. B-scan ultrasound revealed point-like echodensities in the posterior vitreous cavity [Figure 5]. On optical coherence tomography (OCT), optically dense particles were noticeable on the surface of the retina. On follow-up, 26 months after proton beam radiation, best-corrected visual acuity was 20/400, IOP was 30 mm Hg, there was progression of the vitreous and epiretinal pigmentation [Figure 6], and the epiretinal deposits noted on OCT had coalesced into a thick and dense epiretinal membrane in the macular area [Figure 7]. After analyzing the funduscopic and OCT results, a vitreous seeding caused by ciliary body melanoma and the malignant epiretinal membrane was suspected. To confirm the diagnosis, a vitreous biopsy was recommended. However, the patient chose not to proceed with the biopsy and instead opted for enucleation of the right eye, which was performed 29 months after proton beam radiation on her request. Histopathological examination of the enucleated globe showed a necrotic ciliary body melanoma compatible with the treatment effect, and an adjacent focal area of residual tumor. Numerous pigmented macrophages were present in the anterior chamber angle. There were visible pigmented melanoma cells in the vitreous cavity, and a sheet-like growth of viable spindle melanoma cells was also present on the retinal surface [Figure 8]. There was no extraocular extension. The patient has been followed for 10 months after enucleation without any evidence of orbital tumor recurrence or distant metastasis.

##  DISCUSSION 

Clinically detectable vitreous and epiretinal seeding by uveal melanoma has rarely been reported. In a recent case report and review of the literature, Raval et al found 39 reported cases of vitreous seeding.^[3]^ In 10 eyes, the diagnosis of vitreous seeding was made before treatment, and in the other 29 cases, it was made after plaque radiotherapy (22 eyes), proton beam radiation (4 eyes), transpupillary thermotherapy (1 eye), and transscleral resection (2 eyes). In the 22 eyes treated with plaque radiotherapy, the mean interval between treatment and the first appearance of vitreous seeding was 2.3 years.^[3]^

Vitreous seeding from choroidal melanoma is generally limited to tumors with overlying Bruch membrane rupture (usually mushroom-shaped tumors) that subsequently invade the neurosensory retina and seed the vitreous cavity.^[4]^ Uveal melanomas originating in the ciliary body, which is the case in our patient, do not have an overlying Bruch membrane and can gain access to the vitreous cavity after invasion of the two-layered ciliary epithelium.

In our opinion, most eyes with clinically visible vitreous pigment do not require any immediate diagnostic or therapeutic intervention. There are two reasons for this belief: 1) many instances of vitreous pigment in eyes with uveal melanoma represent benign macrophages containing melanin released from the necrotic melanoma rather than malignant melanoma cells;^[1]^ 2) based on available data, the development of vitreous/epiretinal seeding by malignant melanoma cells does not seem to portend a higher risk of distant metastasis.^[1,4]^ It is, therefore, our approach to observe eyes with relatively mild vitreous pigment for evidence of progression. We generally recommend a vitreous biopsy if: 1) there is a definite progression of the vitreous/epiretinal pigment on funduscopy; and 2) OCT shows the development of discrete seeds on the surface of the retina. OCT evidence of coalescence of the epiretinal seeds into an epiretinal membrane or invasion of the neurosensory retina by the epiretinal seeds/membrane should be considered an important sign in favor of a malignant epiretinal membrane.

Our patient had an increased IOP at a presentation that was assumed to be secondary to obstruction of the trabecular meshwork with pigment-laden macrophages, a condition referred to as melanomalytic glaucoma.^[5]^ The increased IOP could have been, at least partially, caused by obstruction of the trabecular meshwork with malignant melanoma cells, but no viable melanoma cells were seen in the angle on histopathologic evaluation after enucleation.

In summary, we report histopathologically documented vitreous/epiretinal seeding in an eye with ciliary body melanoma treated with proton beam radiation. Despite the regression of the original tumor, there was progressive seeding of the vitreous cavity and retinal surface culminating in the formation of a malignant epiretinal membrane. This case underlines the importance of regular monitoring of vitreous pigment in eyes with uveal melanoma, even when the treated tumor itself shows signs of regression on clinical examination. In addition to clinical findings, certain OCT features can be helpful in alerting treating physicians to the development of this uncommon but serious condition.

##  Financial Support and Sponsorship

Support provided in part by an unrestricted educational grant from the Marco Family Foundation.

##  Conflicts of Interest

None.
